# Effect of Argon Plasma Treatment on Tribological Properties of UHMWPE/MWCNT Nanocomposites

**DOI:** 10.3390/polym8080295

**Published:** 2016-08-11

**Authors:** Nitturi Naresh Kumar, Seong Ling Yap, Farah Nadia Dayana bt Samsudin, Muhammad Zubair Khan, Rama Sreekanth Pattela Srinivasa

**Affiliations:** 1 Plasma Research Laboratory, Department of Physics, Faculty of Science, University of Malaya, 50603 Kuala Lumpur, Malaysia; nitturinaresh@gmail.com (N.N.K.); fndsam88@gmail.com (F.N.D.S.); 2 Department of Physics, Federal Urdu University of Arts, Science & Technology, 45320 Islamabad, Pakistan; mzk_qau@yahoo.com; 3 Department of Mechanical Engineering, National Institute of Science and Technology, 761008 Berhampur, India

**Keywords:** UHMWPE/MWCNTs nanocomposites, UHMWPE, biomaterials, dielectric barrier discharge (DBD), surface modification, wear resistance

## Abstract

Ultra-high molecular weight polyethylene (UHMWPE) is widely used in artificial joints in the replacement of knee, hip and shoulder that has been impaired as a result of arthritis or other degenerative joint diseases. The UHMWPE made plastic cup is placed in the joint socket in contact with a metal or ceramic ball affixed to a metal stem. Effective reinforcement of multi-walled carbon nanotubes (MWCNTs) in UHMWPE results in improved mechanical and tribological properties. The hydrophobic nature of the nanocomposites surface results in lesser contact with biological fluids during the physiological interaction. In this project, we investigate the UHMWPE/MWCNTs nanocomposites reinforced with MWCNTs at different concentrations. The samples were treated with cold argon plasma at different exposure times. The water contact angles for 60 min plasma-treated nanocomposites with 0.0, 0.5, 1.0, 1.5, and 2.0 wt % MWCNTs were found to be 55.65°, 52.51°, 48.01°, 43.72°, and 37.18° respectively. Increasing the treatment time of nanocomposites has shown transformation from a hydrophobic to a hydrophilic nature due to carboxyl groups being bonded on the surface for treated nanocomposites. Wear analysis was performed under dry, and also under biological lubrication, conditions of all treated samples. The wear factor of untreated pure UHMWPE sample was reduced by 68% and 80%, under dry and lubricated conditions, respectively, as compared to 2 wt % 60 min-treated sample. The kinetic friction co-efficient was also noted under both conditions. The hardness of nanocomposites increased with both MWCNTs loading and plasma treatment time. Similarly, the surface roughness of the nanocomposites was reduced.

## 1. Introduction

Ultra-high molecular weight polyethylene (UHMWPE) is a successful biomaterial used as an acetabular cup in hip joint replacement. Its unique characteristics are ease of fabrication, high impact strength, low friction coefficient, excellent toughness, biocompatibility, and biostability. When this biomaterial is implanted it undergoes a sequence of critical tasks, such as shock protection/absorption and rotational strains. In this process, because of poor wear resistance from UHMWPE liners, there is a high possibility to generate sub-micron wear debris, as this is the weakest material with counter-face femoral heads in artificial joint implants. Wear results in reduction of the life span for the implant and induces a revision within less than 10 years of primary arthroplasty. Since it is the weakest part in prosthetic joint implants, it has attracted many research groups to further extend the service life of joint prostheses, particularly for younger and more active patients [1].

Researchers have made numerous attempts on improving the wear resistance property of UHMWPE by the use of traditional surface modification techniques, like chemical treatment [2], vitamin E-doping [3], irradiation cross-linking [4], gamma irradiation [5], ion implantation [6], photons [7], and laser impingement [8]. Two of the main issues with these techniques are that they use expensive machinery and require high safety precautions. On the other hand, research attempts were done to enhance the mechanical properties of UHMWPE by reinforcing with micro- or nano-sized particles, thereby improving wear resistance. Different reinforcements, such as carbon nanotubes [9], carbon fibers [10], hydroxyapatite [11], zirconium [12], titanium particles [13], natural coral [14], alumina [15], zinc oxide [16], and kaolin [17] were used as fillers. All observations from these studies showed positive effects of reinforcement. Reinforcements such as carbon fibers, carbon nanotubes, hydroxyapatite, zinc oxide (ZnO), etc., also confirmed significant improvements in mechanical properties of UHMWPE which, in turn, increases the wear resistance. In addition, the surface micro-hardness of UHMWPE increased with reinforcement of carbon nanotubes (CNTs) or carbon fibers, and subsequently leads to improvement in abrasion resistance. Although researchers proved significant improvements in mechanical and wear properties, UHMWPE is still limited and cannot be used of its non-polar nature. 

Many studies have shown that by substantial changes in the polymer surface, affects the chemical and electronic properties, wettability and surface energy, all of which can be achieved by non-thermal plasma treatment. In the past years, studies were done using plasma technique, active screen plasma nitriding, to modify the surface of UHMWPE [18]. It was found that plasma treatment resulted in the formation of OH groups on the treated surface and enhanced wettability, biocompatibility and lubrication during the contact in physiological environment. It is known that –NH_2_, –OH, –CHO, and –COOH groups on polymer surfaces can produce adhesion of cell material with an adequate balance between electron-accepting and electron-donating characteristics of interfaces [19]. Fixation of such groups on inert polymer surfaces like polyethylene, polypropylene, polyvinyl chloride, and UHMWPE is cumbersome. There are potential interests of industry to apply UHMWPE with its inert chemical nature for bio-medical applications, biosensors, and others such as orthopedic implants, especially for load-bearing applications [20]. The structural changes in the polymer surface caused by plasma ions could induce improvements in hardness, wettability, surface chemical activity, cross-linking and other properties. As compared with other surface modification techniques, plasma treatment is a very fast, clean, environmentally safe, and an effective way to modify the polymer surface [21]. Furthermore, the surface properties of treated materials are enhanced eventually while the bulk properties remain unchanged. In addition, to these advantages the treated material is sterilized free from micro-organisms [20]. 

Here we report our experimental findings from plasma treatment on UHMWPE/MWCNT nanocomposites. The treatment was achieved by modifying the alternating current (AC) glow discharge system into a dielectric barrier discharge (DBD) configuration by using glass as a dielectric material [22]. The main reason behind the design modification is that DBD offers a non-equilibrium plasma generated at atmospheric pressure, providing an effective tool for surface activation. DBDs are often filamentary, depending on operating conditions and that can affect the uniformity of the treated polymer surface. An applied voltage of a few kV at frequencies between 5 and 500 kHz is sufficient to create the active discharge region. The present study focuses on the structural changes on the surface along with the wear properties of treated nanocomposites. Properties, such as surface roughness and kinetic friction coefficient, were assessed at the end of the steady-state period. The hardness of the samples was also estimated.

## 2. Experimental Materials and Methods

### 2.1. Materials

The UHMWPE (GUR 1020) in powder form was obtained from Ticona GmbH, Frankfurt, Germany. The molecular weight is 4 × 10^6^ g/mol with an average particle size of 140 ± 20 µm and the density of 0.93 g/cc. The MWCNT was purchased from Shenzhen Nanotech port Co., Ltd., Shenzhen, China. The specifications of as received MWCNTS are as follows: outer diameter 40–60 nm, length 5–20 µm, purity 95 wt %, ash content < 1.5%, density 2.16 g/cc, specific surface area > 200 m^2^/g.

### 2.2. Preparation of Nanocomposites

The nanocomposite preparation was explained in detail in our previous work [23]. The MWCNTs were chemically treated as described in [24] to ensure homogenous dispersion in the polymer matrix. The required sample of MWCNTs were suspended in the mixture of concentrated nitric acid and sulfuric acid at a volume ratio of 1:3 and boiled at 140 °C for 140 min. The sample was dried in a hot air oven at 100 °C. In the next step, UHMWPE was physically blended with chemically-treated MWCNTs at different concentrations, such as 0.5, 1.0, 1.5, and 2.0 wt %. A ball milling machine (The Planetary Mono Mill PULVERISTETTE 6, Fritsch, Cranbury, NJ, USA) was used to prepare nanocomposites. The ball mill time was optimized at 45 min [25]. The obtained raw material mixture was then proceeded in a compression molding machine, Saumya, Ahmedabad, India, 25 ton capacity, at a molding pressure of 10 MPa with the temperature of the upper and lower heating plate at 230 and 220 °C, respectively, maintained for 60 min. The mold was then allowed to cool to room temperature by circulating water into the dies. The obtained nanocomposite sheet is of 0.5 mm thick and 20 × 20 cm^2^ cross-section. To facilitate plasma treatment for the obtained nanocomposite samples, the test samples were cut from a sheet in a cylindrical pill-like shape 10 mm in diameter.

### 2.3. Plasma Treatment

**Figure 1:**
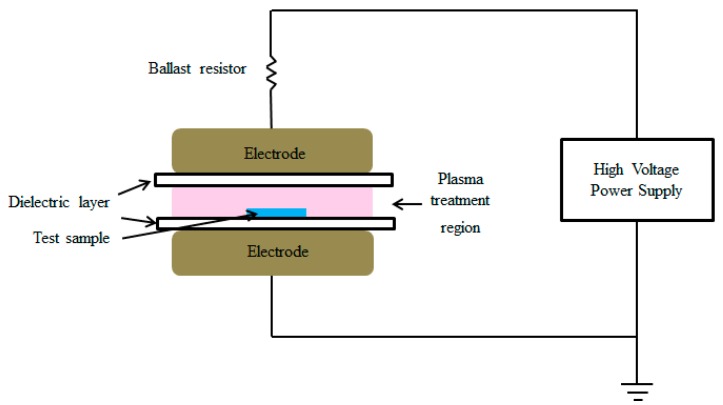
Schematic diagram of DBD for plasma treatment of test sample.

The nanocomposite sheets were cleaned ultrasonically with acetone and absolute ethanol for 5 min., and dried in a vacuum drying chamber at 40 °C for 24 h before plasma treatment. Argon plasma was generated in the parallel plate dielectric barrier discharge reactor. Argon gas, was flowed into the chamber with the working pressure maintained at 18.5 mbar. A 50 Hz alternating current high voltage is applied to the electrodes through a ballast resister of 4 MΩ. The maximum peak to peak voltage of 15 kV. In the dielectric barrier discharge (DBD) configuration (Figure 1) a fairly even discharge was obtained. The parallel plate electrodes were made by brass, with diameter 80 mm and thickness 6 mm. The gap between the dielectrics layers was 3 mm, with dimensions 100 mm × 100 mm × 1 mm, is placed to establish the system in the DBD configuration. The nanocomposites samples were placed freely on the surface of the bottom dielectric layer for plasma treatment. Four different treatment time intervals5, 10, 30, and 60 min were applied on each of the nanocomposites of 0, 0.5, 1.0, 1.5 and 2.0 wt %.

### 2.4. Surface Analysis

The untreated and treated nanocomposites samples were analysed. The surface wettability was estimated by static contact angle measured by the sessile drop method in an ambient atmosphere before and after the DBD plasma treatment, using a JY-82 contact angle tester (Attension theta lite, Biolin Scientific, Stockholm, Sweden) with double deionized water. The instrument was equipped with a charge-coupled device (CCD) camera to capture the contact angles created at the liquid/solid interface. A microsyringe was used to dispense 5 µL drops onto the test sample. Three measurements at different regions on the surface of each nanocomposites samples were obtained.

The presence of possibly new chemical groups on the surface of treated nanocomposites was determined by Fourier Transform Infrared (FTIR) spectrometry (Nicolet Nexus 8700, Waltham, MA, USA); in the range of 4500 to 500 cm^−1^, accumulating 64 scans at a resolution of 4 cm^−1^. A background scan was conducted prior to taking the measurements and was subtracted from each sample spectra. 

### 2.5. Wear Studies Using a Pin-on-Disc (POD) Tribometer

The wear tests of composites, both virgin and plasma-treated, were performed using a POD Tribometer, (Model: TR-20, Ducom Instruments (Asia), Bangalore, India). The sample used was a circular pellet of 4 mm diameter, which was punched from the plasma-treated samples. The test specimen was safely secured at the end of the pin. The test was performed under a load of 30 N at a sliding speed of 0.3 m/s on track diameter of 100 mm for a total sliding distance 1000 m against a 316L stainless steel disc as a counterface material with a surface roughness of 0.1 µm. The UHMWPE material was intended to be used as an articulating surface in total joint replacement. Therefore, assuming a man of 60 kg on an acetabular cup of 28 mm diameter, the pressure was calculated to be 0.974 MPa. Assuming a factor of safety of 2.5, a pressure of 2.43 MPa should be bearable. Thus, in order to produce the same pressure in the circular pellet of 4 mm diameter, the load was found to be 30 N. The test was performed until a steady-state condition was reached, which was noted to be much below 1000 m sliding distance. The linear wear of the sample was monitored by a linear variable differential transformer (LVDT) probe attached to the pin-on-disc tribometer. Since the contact angle influences the wettability of the composites, which in turn strongly affects the wear behavior of test samples, the wear studies were also performed under lubrication conditions using normal saline (N-Saline). The wear factor and friction coefficient are summarized after attaining the steady-state condition. 

### 2.6. Hardness and Surface Roughness Measurements

The microhardness measurements were obtained with a Vickers hardness tester (Tech-ED Equipment’s, Kolkata, India). The Vickers hardness test has been considered a valid tool for evaluating the hardness, and other responses of rigid polymers. The indenter is a regular tetragonal pyramid diamond tip with a vertex angle of 136°. The hardness was measured under a 100 g load with a holding time of 30 s. The surface roughness (*R*_a_, µm) was analyzed using a surface roughness tester (Mitutoyo SJ210, Kawasaki, Kanagawa, Japan), both in dry and wet conditions, at the end of the steady-state. 

## 3. Results and Discussion

### 3.1. Water Contact Angle Measurement

**Figure 2:**
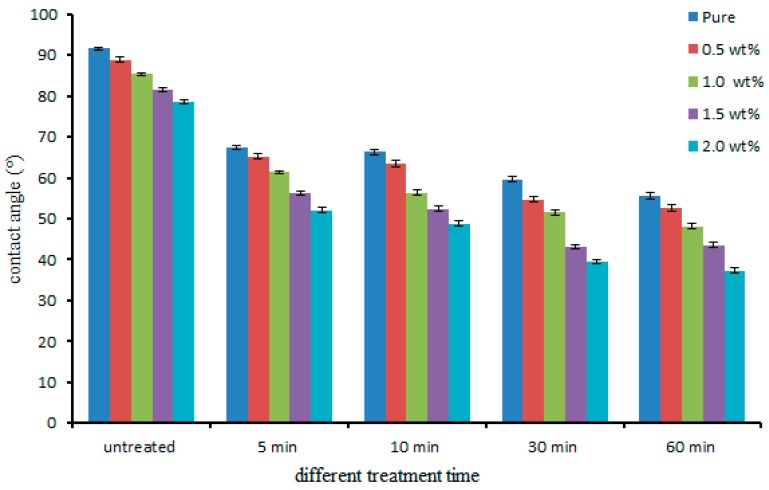
Wettability of nanocomposites before and after plasma treatment at different time exposures.

The water contact angles measured for untreated and all the plasma treated nanocomposites samples are shown in Figure 2. The average values of four repeated measurements of untreated samples are 91.1°, 88.4°, 85.4°, 81.2°, and 78.1° for pure, 0.5, 1.0, 1.5, and 2.0 wt % of MWCNT composites, respectively. It is observed that UHMPWE with increasing concentration of MWCNTs shown there is a slight decrease in contact angle. This could be due to the fact that the MWCNTs were acid-treated for more than 24 h before used as reinforcement. Recent studies have shown that the surface energy of the 24-h acid functionalized multi-walled carbon nanotubes/poly(methyl methacrylate) (MWCNTs/PMMA) composite increased by approximately 2.13 times compared to that of virgin PMMA, as evaluated by the Fowkes method [26]. Apart from this, functionalization also introduces chemical groups, carboxyl (–COOH) and hydroxyl (–OH), which directly influence adhesion at the liquid and solid interface. Although studies from other groups reported similar values for pure UHMWPE none, as per the author’s knowledge, have reported the values of contact angles for UHMWPE/MWNCT nanocomposites.

Significant reduction in contact angles was obtained with plasma treatment of 5 min or longer. The contact angles measured 67.4°, 64.0°, 60.5°, 55.5°, and 51.4° for pure, 0.5, 1.0, 1.5, and 2.0 wt % of MWCNTs, respectively after 5 min of plasma treatment Longer exposure for 60 min duration led to even more decreased values of the water contact angle. If the contact angle is between 0° and 30° it indicates a highly hydrophilic solid, where the liquid is completely spread out on the solid surface [27]. In the present case, it has been observed that the contact angle of 60 min-treated, 2.0 wt % sample, is 37.1°, which is approximately a 50% reduction as compared to untreated samples. In general, the results indicate that the MWCNT reinforcement coupled with plasma treatment could make the UHMWPE surface highly hydrophilic, which is greatly desirable for tribological application in load-bearing joints. 

### 3.2. ATR-FTIR Spectrometry

**Figure 3:**
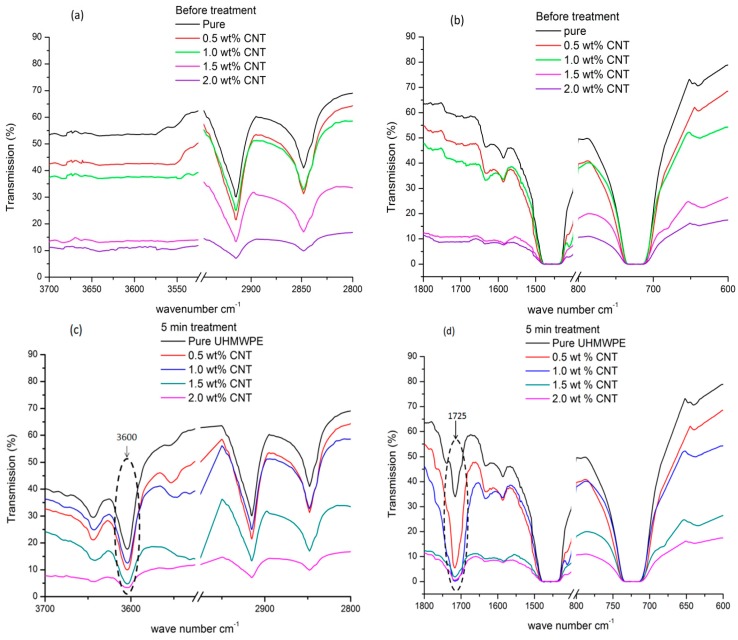
FTIR-ATR spectra of before plasma treatment (**a**) and (**b**); 5 min plasma-treated sample (**c**) showing 2850, 2920, and 3600 peaks; and (**d**) showing 722, 1465, and 1723 peaks.

The above results indicate that the wettability of nanocomposites have been improved significantly by plasma treatment. The result of polar groups such as carbonyl and hydroxyl groups formed on the treated surface are confirmed by Fourier transform infrared spectroscopy (FTIR) It is well known that UHMWPE is a type of polyolefin, so the characteristic absorption peaks attributed to the varied vibration of methylene are more typical and obvious, and which includes the stretching (between 2975 and 2845 cm^−1^), the scissoring (between 1470 and 1430 cm^−1^), and in-plane rocking (between 780 and 680 cm^−1^). These peaks have been detected at all treatment times i.e., 5, 10, 30, and 60 min. The FITR spectrums for 5 min plasma-treated nanocomposite and non-treated nanocomposite samples are shown in Figure 3.

After plasma treatment, the absorption peaks of 2917, 2848, 1467, and 722 cm^−1^ are attributed to methylene non-symmetry stretch vibration, methylene symmetry stretch vibration, methylene non-symmetry changing angle vibration, and methylene swing in-plane vibration, respectively. Absorption peaks at 2917 and 2848 cm^−1^ indicate the C–H asymmetric and C–H symmetric stretching vibrations in –C–H_2_ respectively. Another small absorption peak in the IR spectrum at 1467 cm^−1^ can also be attributed to C–H vibration deformation, while the peak at 722 cm^−1^ is due to C–C rocking vibrations in –(CH_2_)_n_–. A new broadband observed around 3600 cm^−1^, see Figure 3c, on treated samples which correspond to the hydroxyl group (–OH), broadening can be due to its intermolecular or intramolecular reactions [28]. The broadband around 1725 cm^−1^, see Figure 3d, is also observed in the spectrum, which corresponds to various oxygen containing (C=O stretch) polar functional groups. These additional peaks show the post-treatment functionalization of the nanocomposites surfaces. 

### 3.3. Wear Studies on Plasma-Treated Samples

#### 3.3.1. Wear Studies of Plasma-Treated Samples under Dry Conditions

**Figure 4:**
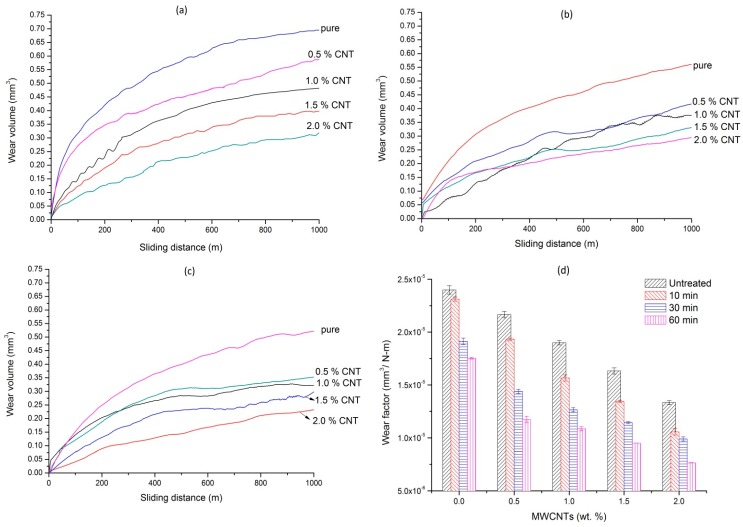

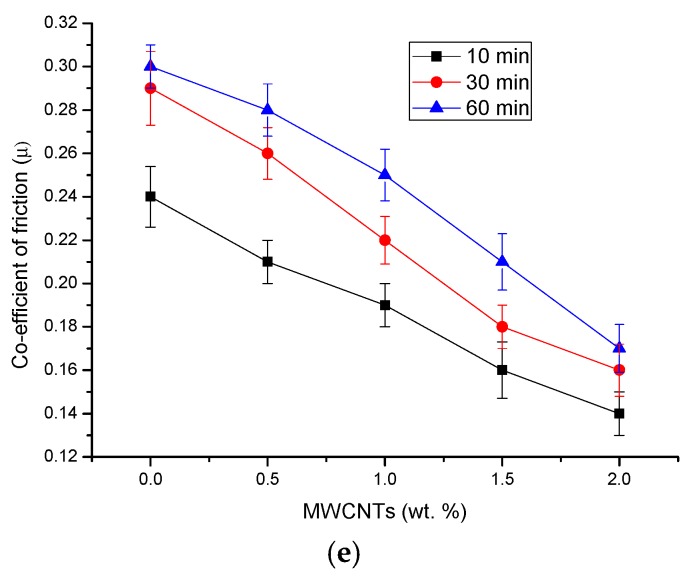
The sliding distance vs. wear volume of plasma treated samples (**a**) 10 min; (**b**) 30 min; (**c**) 60 min; (**d**) wear factor (**e**) coefficient of friction under dry test conditions.

The argon plasma treated nanocomposites samples were tested for wear behavior under dry and wet conditions. The wear volume of plasma-treated composites at different time intervals of 10, 30, and 60 min are shown in Figure 4a–c, respectively. It is observed that the wear volume of a 10 min treated pure UHMWPE was found to be 0.694 mm^3^ at the end of the predefined sliding distance of 1000 m. The wear volume was further reduced to 0.58, 0.47, 0.40, and 0.31 mm^3^, which corresponds to a reduction of 15.9%, 31.8%, 42%, and 55%, respectively for 0.5, 1, 1.5, and 2 wt % MWCNT nanocomposites. It is observed that the wear volume of untreated pure UHMWPE sample reduced from 0.72 mm^3^ to 0.69, 0.574, and 0.526 mm^3^ after plasma treatment for 10, 30, and 60 min, respectively. Similarly, for untreated 2 wt % composite, the wear volume was reduced from 0.4 mm^3^ to 0.31, 0.29, and 0.23 mm^3^ after plasma treatment for 10, 30, and 60 min, respectively. Based on the above results it is noted that the wear volume reduced with an increase in MWCNT concentration and also with plasma treatment time. The reason for such reduction of wear volume is two-fold; firstly, the incorporation of MWCNTs; and secondly, the structural changes in the surface of UHMWPE/MWCNTs nanocomposites due to argon plasma surface modification as confirmed by the FTIR studies. 

MWCNTs are known for their superior mechanical properties [29] where the increased the hardness of the composites are due to their increased crystallinity, which results in the formation of harder and more rigid polymer and thus better were resistance. Increased hardness in epoxy [30], PMMA [31], and UHMWPE [32] of 30%, 50%, and about 12%, respectively, by the reinforcement of MWCNTs have been reported. More to it, the plasma treatment induces surface crosslinking and surface oxidation, which was reflected through FTIR studies. The increased crosslinking increases the surface crosslink density of the plasma-treated samples [33]. The toughness of the polymer is directly related to its crosslink density which, in turn, influences the wear resistance. Apart from this, the shear strength of UHMWPE surface also increases due to crosslinking, thus restricting the material removal from the bulk. In addition to this, the literature [34] also reports that post-treatment functionalization occurs on the surface of plasma-treated samples resulting in the oxidation of the surface leading to surface hardening and, thus, offers superior wear resistance.

The wear factor, which is defined as the volume of material removed per unit work done, is representative of the wear resistance of the material and is shown in Figure 4d. The wear factor of pure untreated UHMWPE was found to be 2.4 × 10^−5^ mm^3^/N-m, compared to 7.6 × 10^−6^ mm^3^/N-m for 2 wt % 60 min-treated sample under dry sliding conditions. The coefficient of friction of the test samples against MWCNTs concentration at different treatment periods is shown in Figure 4e. It is observed that the coefficient of friction was reduced with MWCNTs concentration; however, it was increased with plasma treatment time. The reduction in the friction coefficient with an increase in MWCNT concentration can be attributed to the lubricating nature of carbon nanotubes, whereas increased plasma treatment time resulted in a thicker coating, one that which was rough in nature. In order to confirm this, the surface roughness of the treated samples was measured using Mitutoyo Talysurf. The surface roughness (*R*_a_) of untreated polymers and composites was found to be 0.25 µm, which increased to 0.28, 0.31, and 0.35 µm for 10, 30, and 60 min in plasma treated samples. A similar observation was reported [35], on plasma treated UHMWPE where the increase was observed to be two-fold compared to untreated sample .

**Figure 5:**
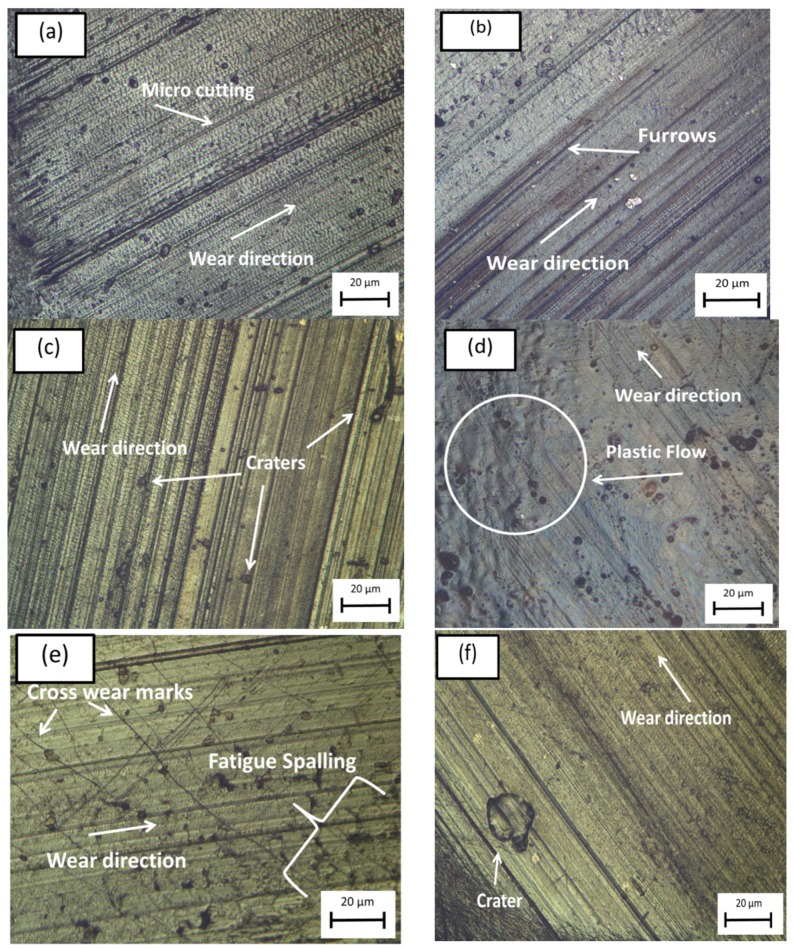
Optical microscope images of test samples (2 wt % UHMWPE/MWCNT nanocomposites with 60 min plasma treatment) under dry sliding conditions showing different wear mechanisms (**a**) microcutting; (**b**) furrows; (**c**) craters; (**d**) plastic flow and deformation; (**e**) cross-wear marks and fatigue spalling; and (**f**) wear crater.

The nanocomposites samples with different concentration of WMCTs subjected to the wear test in dry condition showed similar wear behavior. A set of the images showing the physical appearance of 2% wt are shown in the Figure 5. The wear direction is shown in each of the images and is indicated with an arrow. Figure 5a shows the microcutting resulting due to the abrasive action of wear generated particles, severely cutting the polymer surface. A few wear craters are also observed in the same image. Figure 5b,c shows the furrows and craters. The plastic flow was shown in Figure 5d, resulted because of frictional heating, leading to subsequent deformation and wrinkle formation on the test sample. Figure 5e shows the cross-wear marks, which could be due to third body wear. The cross-wear marks are seen to cross the prominent wear furrows generated due to the wear direction. Fatigue spalling is also depicted, which would be formed due to repeated loading and plastic deformation, [36]. Figure 5f shows a typical wear crater observed in the 2 wt % nanocomposite with 60 min plasma treatment. Similar wear mechanisms were observed in all the samples in different intensity.

#### 3.3.2. Wear Studies of Plasma Treated Samples under N-Saline Lubricant Conditions

**Figure 6:**
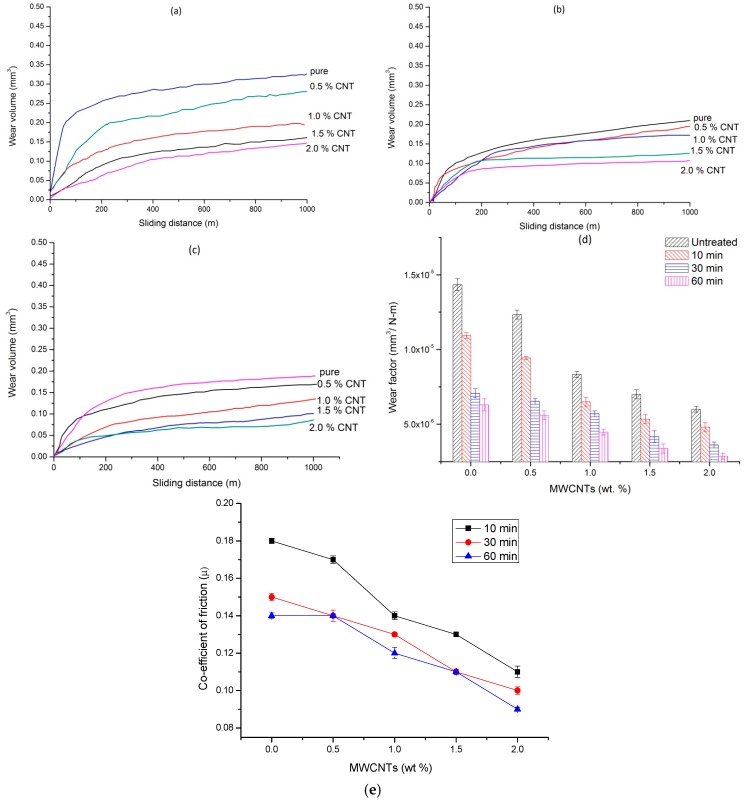
The sliding distance vs. wear volume of plasma-treated samples (**a**) 10 min; (**b**) 30 min; (**c**) 60 min; (**d**) wear factor; and (**e**) coefficient of friction, under N-Saline lubricant conditions.

Tribology studies were performed under N-saline lubricant conditions. Figure 6a–c shows the wear volume of nanocomposites subjected to plasma treatment for 10, 30, and 60 min, respectively. The wear volume of a 10 min-treated pure, 0.5, 1.0, 1.5, and 2 wt % MWCNT nanocomposites were found to be 0.328, 0.283, 0.195, 0.16, and 0.144 mm^3^, respectively; while the same for a 60 min-treated sample, the corresponding wear volumes were found to be 0.189, 0.168, 0.134, 0.101, and 0.086 mm^3^. It is noted that the wear volume of a 60 min-treated, 2 wt % nanocomposite was reduced by 73.7% compared to a 10 min treated pure UHMWPE. The wear volume of nanocomposites under lubrication conditions was found to be considerably less compared to dry wear conditions. The wear volume of a 60 min-treated, 2 wt % nanocomposite was found to be 0.23 mm^3^ under dry conditions, was reduced to 0.086 mm^3^ upon lubrication, which corresponded to a 62% reduction. 

A similar trend was observed in the case of all of the nanocomposites treated at different periods of plasma treatment. The wear factor of the test samples for all the conditions of plasma treatment are shown in Figure 6d. The trend observed was similar to that of dry conditions, i.e., wear factor was reduced in both the conditions and was increased with MWNCT concentration and plasma treatment time. It could also be observed that the wear factor of a 60 min-treated pure UHMWPE was 6.3 × 10^−6^ mm^3^/N-m, while for a 2 wt % sample, was 2.86 × 10^−6^ mm^3^/N-m. This denotes that the wear factor of untreated virgin UHMWPE was reduced by 56% due to the effect of a 60 min plasma treatment and 80% due to the combined effect of 2 wt % MWCNT reinforcement and plasma treatment. Figure 6e shows the coefficient of friction of the wear samples at different periods of plasma treatment. The coefficient of friction was reduced with an increase in MWCNT concentration and also plasma treatment time; the friction coefficient of a 10 min-treated pure UHMWPE decreased from 0.18 to 0.14 upon increasing the treatment time to 60 min, and which was further reduced to 0.09 with reinforcement of 2 wt % MWCNTs into UHMWPE. This trend is unlike that which was observed under dry test conditions, where the coefficient of friction increased with plasma treatment time. The reason for this is that, with the increase in the plasma treatment time, the contact angle was reduced. This increases the surface wettability of the test specimen and, thus, eliminates the chances of excessive contact with the counterface forming a thin layer of lubricant. The layer of lubricant would, therefore, ensure a much smoother relative motion between the nanocomposite sample and the stainless steel counterface, thus reducing both wear volume and friction coefficient. An important observation is that the friction coefficient of 60 min-treated, 2 wt % nanocomposite is 0.09, which is much closer to the friction coefficient value of natural joints (0.03).

**Figure 7:**
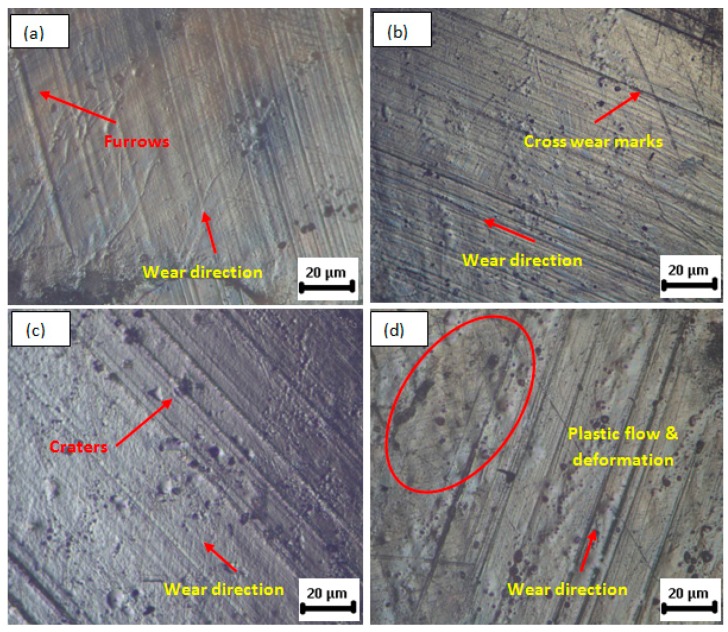
Optical microscope images of test sample (2 wt % UHMWPE/MWCNT nanocomposites with 60 min plasma treatment) under N-Saline lubricant sliding conditions. (**a**) Furrows and wear direction; (**b**) cross-wear marks; (**c**) craters; and (**d**) plastic flow and deformation.

The surface morphology of the 2 wt % nanocomposites with 60 min plasma treatment, showed several wear mechanism in the wet conditions are shown in Figure 7a–d. Wear furrows are seen in Figure 7a, with mild occurrence of wear craters. Figure 7b shows the cross-wear marks observed, and showed that the severity of the cross-wear marks was less in wet conditions compared to dry conditions. Cross-wear marks are usually caused by third wear, resulting from the trapped wear particles between the surfaces of relative motion. In the case of lubricated condition, the entrapped wear particles would be carried away by the lubricant thus reduce the formation of cross-wear marks. The same can be observed with other wear phenomena such as craters and plastic flow as shown in Figure 7c,d, respectively. The plastic flow due to the frictional heating was also observed to be reduced in its intensity, as the heat was carried away by the lubricant. Fatigue spalling was not at all observed in the lubricant conditions. It is inferred that argon plasma treatment and MWCNTs reinforcement into UHMWPE would enhance its wear resistance by increasing the surface hardness and surface wettability. The wear resistance would be further enhanced under lubricated conditions, indicating an improved life of the articulating materials used in joint replacement.

### 3.4. Hardness and Surface Roughness of Nanocomposites

**Figure 8:**
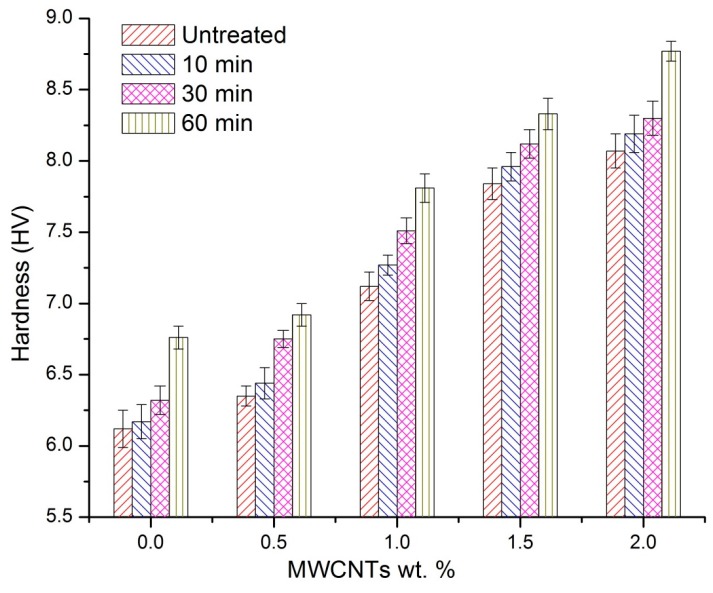
Variation of hardness of UHMWPE/MWCNTs nanocomposites under different treatment times.

The hardness of the surface is one of the direct parameters that influence the wear of the test samples. The hardness of nanocomposites samples increased with MWCNT concentration and plasma treatment time, as shown in Figure 8. The increased hardness can be attributed to the presence of MWCNTs, which results in an increased crystallinity [37]. Plasma treatment time, as indicated earlier, enhances the interaction and bonding between polymers, leading to MWCNTs and ions forming a harder surface due to the attached functional groups. It is observed from the figure that the hardness of pure UHMWPE is 6.02 (HV), which increased to 8.77 (HV) for a 60 min-treated, 2 wt % composite, corresponding to a 45% enhancement. The increased surface hardness results in an improved wear resistance of the nanocomposites samples. 

**Figure 9:**
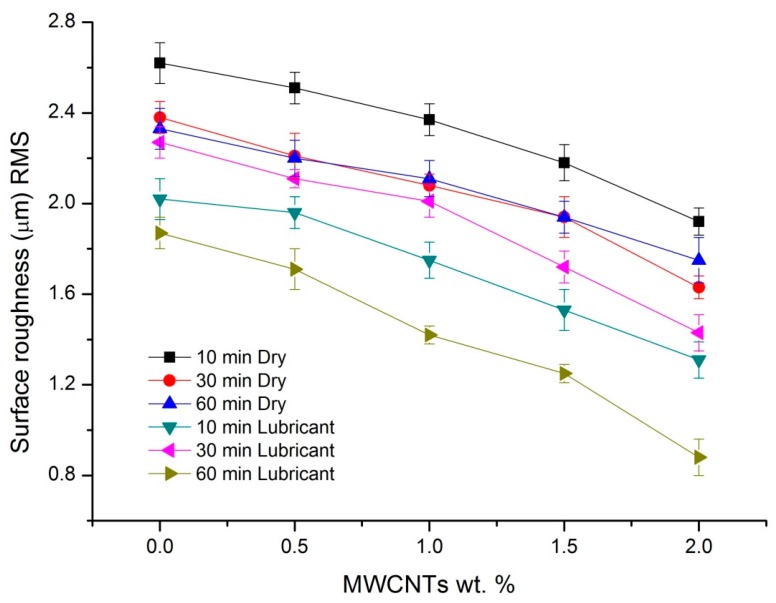
Surface roughness (RMS) value of nanocomposites under dry and lubricant conditions for different treatment times.

The surface roughness of the test specimens is an important parameter, which influences the wear of the sample. Figure 9 shows the Root Mean Square (RMS) value of surface roughness of nanocomposites under dry and wet conditions. It can be observed that the RMS value was reduced with an increase in MWCNT concentration, for both dry and wet test conditions. The surface roughness of pure sample reduced from 2.27 to 1.43 µm (17% reduction) for a 2 wt % sample, under dry sliding conditions; whereas the same, in the case of lubricated conditions, was found to be 1.87 to 1.08 µm (25% reduction).

The reasons for reduced surface roughness could be two-fold: firstly, hardness; the presence of MWCNTs increases the hardness of the samples. A harder sample offers better resistance to scratches which may result due to undulations of the counterface and also due to third-body wear. Plasticity index is another important parameter that influences the surface topography during wear. It indicates that the material for elastic recovery after the deformative load has been removed, i.e., a higher plasticity index indicates a higher plastic nature and less elastic nature. It has been reported elsewhere that the presence of MWCNTs reduces the plasticity index and also softer materials have higher plasticity indexes [38,39]. It is understood that surface roughness resulted by multiple factors, such as third-body wear, counterface scratches, and deformation, all of which result in plastic deformation. However, since MWCNTs reduce the plasticity index, the deformation caused by these factors will also be reduced due to the elastic recovery and, hence, reduced surface roughness. It also is observed from the figure that surface roughness, which was reduced with an increase in plasma treatment time further declined under lubricated conditions. As discussed earlier, contact angle reduced with an increase in both MWCNT loading and treatment time, resulting in increased wettability of the test samples which, in turn, avoid direct contact of the polymer and metal interfaces and, henceforth, lowers the surface roughness.

## 4. Conclusions

The plasma surface treatment on UHMWPE/MWCNT nanocomposites was successfully carried out with a DBD plasma source with different time intervals. The effect of plasma treatment has been investigated using contact angle measurement, FTIR, and tribological studies under dry and lubricated conditions. Observations from the contact angle measurements evident that, by increasing the treating time, the surface of the nanocomposite is transformed from hydrophobic to hydrophilic. The formations of polar groups are confirmed in the FTIR results. Wear characterization indicates that the initial wear rate is significantly decreased due to an increase in wettability on treated nanocomposite surfaces. A comparison of dry wear under a N-saline lubrication condition showed better tribological properties. The hardness, surface roughness, and friction co-efficient of nanocomposites were positively affected by the plasma treatment. It is concluded that plasma treatment, in synergy with MWCNTs reinforcement, would significantly enhance the wear properties and also provide better lubrication.
